# Effect of the Modified Methacrylate-Based Root Canal Sealer in Single-Cone Technique

**DOI:** 10.3390/nano12213722

**Published:** 2022-10-23

**Authors:** Yu Fan, Zheng Wang, Yan Sun, Xiao Guo, Haohao Wang, Hockin H. K. Xu, Suping Wang, Xuedong Zhou, Bolei Li, Lei Cheng

**Affiliations:** 1 State Key Laboratory of Oral Diseases, National Clinical Research Center for Oral Diseases, West China School of Stomatology, Sichuan University, Chengdu 610041, China; 2 Department of Cariology and Endodontics, West China Hospital of Stomatology, Sichuan University, Chengdu 610041, China; 3 Department of Advanced Oral Sciences and Therapeutics, School of Dentistry, University of Maryland, Baltimore, MD 21201, USA; 4 Stomatology Center, The First Affiliated Hospital of Zhengzhou University, Zhengzhou 450052, China

**Keywords:** root canal sealer, dimethylaminododecyl methacrylate, magnetic nanoparticles, single-cone technique

## Abstract

This study aimed to modify EndoREZ with 2.5% dimethylaminododecyl methacrylate (DMADDM) and 1% magnetic nanoparticles (MNP) to study its sealing property, penetration and long-term antibacterial and therapeutic effect in the single-cone technique (SCT) compared with EndoREZ and iRoot SP. Thirty single-root human maxillary premolars were assigned into three groups and obturated with three different root canal sealers by SCT. Every specimen was then scanned using micro-CT to analyze void fraction, and void volumes and confocal laser scanning microscope (CLSM) was used to study the dentin penetration. The long-term antimicrobial effects were tested in vitro before and after aging 1 and 4 weeks by the single-strain *Enterococcus faecalis* biofilm model. In addition, the beagle canine model of apical periodontitis (AP) was utilized to judge and compare the therapeutic effect of three sealers in SCT. The void fraction and void volumes of the modified root canal sealer were not significantly different from iRoot SP (*p* > 0.05) but were lower than EndoREZ (*p* < 0.05). The modified root canal sealant displayed a greater penetration, long-term antibacterial property, and treatment effect than the other groups (*p* < 0.05). This indicated that after being modified with DMADDM and MNP, it showed better performance in SCT.

## 1. Introduction

Apical periodontitis is a bacterial infectious disease that occurs in the pulp and tissues around the apex [1]. At present, the most common treatment method in clinical settings is root canal therapy (RCT), and the key to success is the complete removal of infectious substances in the root canal and tightly filling it [2]. Well-filled root canals promise to provide a three-dimensional seal to prevent bacterial invasion and entomb surviving bacteria [2].

At present, the main clinical root canal filling techniques include cold lateral compaction (CLC), warm vertical compaction (WVC), the and single-cone technique (SCT). The three filling techniques have their advantages and disadvantages. CLC has been considered the gold standard for novel obturation techniques [3]. WVC is the most mainstream filling technique, which has a good sealing effect on apical root, crown, and lateral root canal, and has wide indications [4]. However, CLC and WVC increase the risk of root fracture because of the use of filling pressure [5]. In addition, due to the limitation of the instrument, neither of these two methods is suitable for severely curved or long root canals [6]. The application of SCT has a long history. As early as 1961, Ingle [7] proposed standardized root canal instrumentation and technology, and then SCT came into being. However, due to the large amount of sealer required by SCT to fill the root canals, there are higher requirements for the sealing capability and antibacterial properties. Limited by the available sealers at the time, SCT was not recommended due to numerous studies showing poor sealing performance [8,9]. With the development of the sealing performance of sealants in the last few decades, SCT is increasingly used in clinical treatment due to its low technical sensitivity, short operation time and strong anti-root fracture performance, and it can be used for large-curvature root canals [10]. In recent years, SCT has once again become a clinical and research hotspot.

IRoot SP (Innovative BioCreamix Inc., Vancouver, Canada), a bioceramic sealer, is one of the most common sealants used for SCT with a high success rate [11]. The main components of iRoot SP are calcium phosphate, calcium silicate, zirconia, and calcium hydroxide. With good biocompatibility, IRoot SP is able to promote cell differentiation and induce osteogenesis [12], which can accelerate periapical tissue healing [13]. Furthermore, the bioceramic sealer has high fluidity [14] and a slight expansion of 0.2% in volume after curing [15], which makes it a good seal for SCT and reduces the occurrence of microleakage. In root canal therapy, it is impossible to remove the infectious substances from the root canal completely, and the residual microbes may cause the failure of RCT. *Enterococcus faecalis* is frequently detected in apical periodontitis, with a detection rate of more than 80% in secondary/persistent endodontic infections [16]. Therefore, the antibacterial activity of sealants is crucial to improve the success rate of RCT. Studies [17] have shown that iRoot SP is effective against *E. faecalis*. However, the direct contact test confirmed that fresh iRoot SP had good antibacterial activity against *E. faecalis*, but its antibacterial activity was significantly weakened after 7 days of mixing [18]. High pH is a possible reason for the antibacterial properties of iRoot SP [18], so the antibacterial effect diminishes over time. In addition, there is no pressure in the SCT, so better seal performance can be achieved if the sealer is actively positioned. Therefore, it is necessary to explore more suitable sealants with long-term antibacterial performance and active position for SCT.

EndoREZ (Ultradent Inc., South Jordan, UT, USA) is a common clinical sealer that is methacrylate-resin-based with hydrophilic and dual-cure properties. It has excellent hydrophilicity and fluidity which allows it to flow into accessory canals and dentinal tubules to promote resin tag formation [19]. Dimethylaminododecyl methacrylate (DMADDM) is a novel quaternary ammonium salt (QAS) antibacterial monomer, used as an antibacterial agent due to its contact-killing effect [20]. Previous studies [21,22] have shown that when DMADDM is added to different dental materials, it can exhibit antibacterial effects against bacteria and biofilms without damaging the materials’ properties. Both EndoREZ and DMADDM have double-bond characteristics in the main components’ chemical structures, which can form cross-linked structures under certain conditions. [23]. Magnetic nanoparticles (MNPs) have good biocompatibility and are widely used in medical biology [24]. Due to their magnetic responsiveness, MNPs can conduct directional movement under the action of a magnetic field and enter the biofilm to destroy the biofilm structure [25]. MNPs have been applied in many aspects of the oral field, showing superior performance in tissue engineering, treatment of oral cancer and oral infections [26,27,28]. We found that adding 2.5% DMADDM and 1% MNP to EndoREZ sealer had no significant effect on its apical sealing performance in WVC, and under an external magnetic field, it could increase the dentin penetration (unpublished data). We wonder whether the modified root canal sealer can perform well in SCT compared with iRoot SP. This project aimed to modify EndoREZ with 2.5% DMADDM and 1% MNP and to investigate the effects on the sealing property, penetration, long-term antibacterial property and treatment effect of the modified root canal sealer in SCT.

## 2. Materials and Methods

### 2.1. Study on Material Properties

#### 2.1.1. Filled Root Canal Preparation

Thirty extracted single-canal maxillary premolars without caries, root resorption or fractures were utilized in this study. The crowns of each tooth were sectioned at the level of the cement–enamel junction, then adjusted to a length of approximately 12 mm. The working length was set at 0.5 mm from the apex. All teeth were instrumented to size #40/06 with ProTaper NiTi rotary instruments (Dentsply Maillefer, Baillagues, Switzerland), irrigated with 1.0% NaOCl (Longly, Wuhan, China).

The teeth were randomly assigned into three groups, and then they were filled with three different root canal sealants using SCT. Group 1: EndoREZ (Ultradent Inc., South Jordan, UT, USA). Group 2: iRoot SP (Innovative Bio Creamix Inc., Vancouver, BC, Canada). Group 3: EndoREZ modified with 2.5% DMADDM (DMADDM was previously synthesized and validated [29]) and 1% MNP (Sigma, St. Louis, MO, USA) by agitating for 5 min and light-curing for 10 s. All sealers were prepared by the first experimenter and placed in the same packaging. The root canal filling procedure was performed by another experimenter. The root canal filling procedure was blinded. In group 3, after filling, a circular magnet (Tiansheng, Shenzhen, China) with a 10 mm diameter and 5 mm thickness was placed on the buccal and lingual side 5 mm away from the sample for 5 min in the first experiment. An amount of 0.1% rhodamine dye was mixed into the three different root canal sealants before filling. After obturation, the samples were deposited at 100% humidity for 7 days at 37 °C to allow the sealant to solidify.

#### 2.1.2. Micro-CT Evaluation

Micro-CT (μCT 50, SCANCO Medical AG, Brüttisellen, Switzerland) was used at high resolution, with parameters 90 kVp, 200 μA, 0.5 mm Al/Cu filter, and 13 µm pixel size to scan samples for detecting void volumes. SCANCO Evaluation (SCANCO Medical AG) was utilized to capture and analyze images. After being reconstructed, the specimens were divided into apical (0–4 mm), middle (4–8 mm), and coronal (8–12 mm) to evaluate voids. The internal voids (inside the sealer), the external voids (along the inner wall of the root canal), and the combined voids (in the sealant connected to the canal walls) were analyzed from the reconstructed images.

#### 2.1.3. CLSM Evaluation

Each root was horizontally sectioned using a DTQ-5 low-speed precision-cutting machine (Wei Yi, Laizhou, China) at apical (3 mm away apex), middle (6 mm away apex), and coronal (9 mm away apex) third. All sections were then polished with abrasive papers on a polisher (Struers, Copenhagen, Denmark) to obtain a 1 ± 0.1 mm slice. A confocal laser scanning microscope (OLYMPUS, Japan) was used to acquire the image of samples with excitation by a He/Ne G laser (543 nm) at 4×/10× magnification. The percentage of dentinal tubule penetration was measured according to the methods from McMichael [30].

### 2.2. Study on Antibacterial Activity and Biosafety

#### 2.2.1. Bacteria Species

The State Key Laboratory of Oral Diseases (Sichuan University, Chengdu, China) provided *Enterococcus faecalis* ATCC29212, which was cultured in Brain-Heart Infusion Broth (BHI; Difco, Sparks, MD, USA) in an anaerobic environment (90% N_2_, 5% CO_2_, 5% H_2_) at 37 °C.

#### 2.2.2. Fabrication of Biofilm Specimens

The fabrication of biofilm specimens was following Li’s study [31]. Composite disks were fabricated using a 48-well plate cover as a mold. We applied 20 mg of sealers to each composite disk’s surface and flattened it with a spatula, then incubated them at 100% humidity for 7 days at 37 °C to solidify. After being sterilized in ethylene oxide and without aging or aging for 1 or 4 weeks, each disk inoculated *E. faecalis* (2 × 10^6^ CFUs/mL) at 37 °C anaerobically for 48 h to form biofilms.

#### 2.2.3. Colony-Forming Units (CFU)

Phosphate-buffered saline (PBS) was used to rinse the 48 h biofilms on the disk twice, as the planktonic bacteria could be removed. After scraping and serially diluting the biofilms with PBS buffer, the bacteria were incubated on BHI agar plates and microbial colonies were counted to evaluate their viability.

#### 2.2.4. Scanning Electron Microscopy Detection

After 48 h culture, the biofilms were gently rinsed twice with PBS. They were fixed overnight with 2.5% glutaraldehyde and dehydrated with graded ethanol. Scanning electronic microscopy (FEI, Hillsboro, OR, USA) was used to detect the samples sputter-coated with gold.

#### 2.2.5. Live/Dead Bacteria Staining

After being rinsed twice with PBS, the 48 h biofilms were stained with the BacLight Live/Dead bacterial viability kit (Molecular Probes, Eugene, OR, USA) following the manufacturer’s instructions. A confocal laser scanning microscope (OLYMPUS, Tokyo, Japan) was applied at 40× magnification to detect biofilm images.

#### 2.2.6. Cytotoxicity of Sealant Eluent to Fibroblasts (L929)

The sealant eluent was prepared according to Liu’s study [23]. Sealers were injected into customized rings with 5 mm diameter and 2.5 mm height, which were nonreactive plastic. The samples were set at 100% humidity for 7 days at 37 °C, then sterilized with ethylene oxide. The sealer eluents were obtained by soaking at 37 °C for 24 h in 10 mL Dulbecco’s Modified Eagle Medium (DMEM) with 10% fetal bovine serum, 1% penicillin, and streptomycin.

L929, the fibroblasts, were cultured at 37 °C with 5% CO_2_. The cytotoxicity of the eluent was assessed through the Cell Counting Kit-8 (CCK-8) (DOjinDO, Shanghai, China). Then, a Thermo Scientific Multiskan GO reader was used (Thermo Fisher Scientific Inc., Waltham, MA, USA) to measure the solution absorbance at 450 nm.

### 2.3. The Beagle Canine Model of Apical Periodontitis

The premolars of one 12-month-old beagle dog, weight 8–12 kg, were studied to obtain 18 root canals. The beagle canine model of apical periodontitis was conducted using the methods of Wang et al. [32], who complied with the ARRIVE 2.0 guidelines. The beagle dog was anesthetized with 3% pentobarbital sodium (Aikonchem, Jiangsu, China) with the dosage of 30 mg/kg body weight by intraperitoneal injection to open the pulp cavity. A chronic AP was induced by exposing the canals to the oral environment for 4 weeks. The periapical lesion was observed by cone beam computed tomography (CBCT).

The canals were instrumented to size #25 with ProTaper NiTi rotary instruments (Dentsply, Sirona, York, PA, USA) and irrigated with 1.0% NaOCl. After drying, the root canals were assigned into three experimental groups, filled with three different root canal sealants using SCT, and then the sealed cavity was filled with adhesive resin (3M, St. Paul, MN, USA). After 3 months, the periapical lesion was observed by CBCT. The variation in apical shadow volume was calculated through images reconstructed with MIMICS V21.0. The dog was sacrificed for histopathological examination, and the inflammation grade was classified following Wang et al. [32].

### 2.4. Statistical Analysis

SPSS software version 16.0 (SPSS Inc., Chicago, IL, USA) was utilized for statistical analysis. One-way ANOVA and Kruskal–Wallis analysis were used for comparison at a significance level of α = 0.05. Significant differences were considered when *p* < 0.05.

## 3. Results

### 3.1. Void Fraction and Void Volume

**Figure 1:**
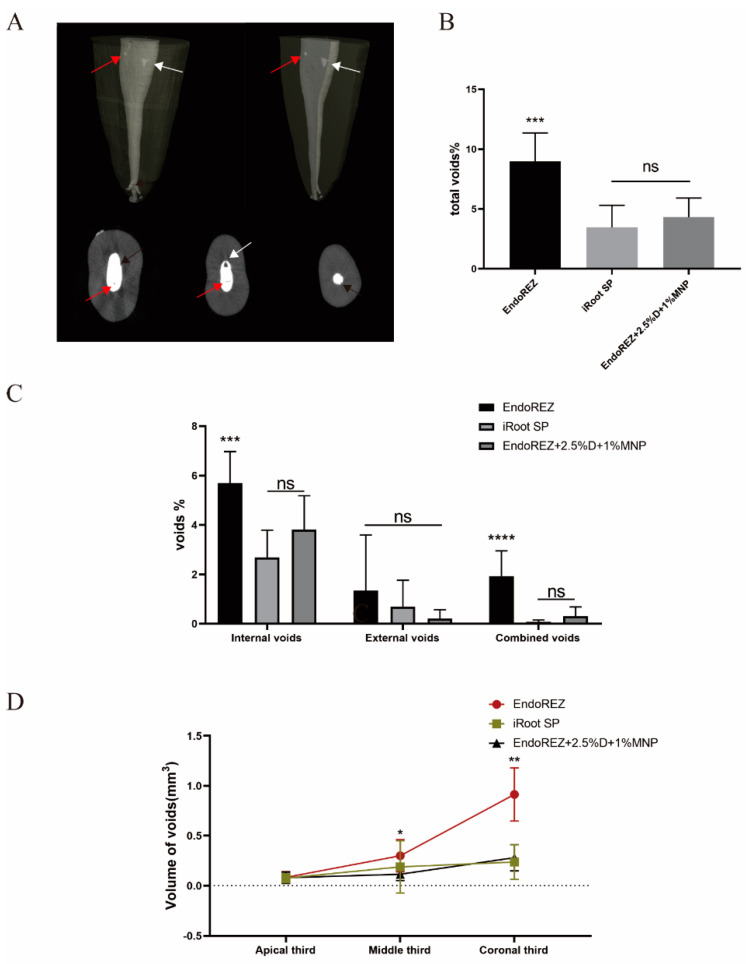
The sealing property of three sealers by micro-CT. (**A**) Two-dimensional slices and three-dimensional reconstruction scanned with micro-CT (voxel size = 13 μm) show the 3D volumes of voids after the root canal treatment. Black arrows indicate external void, red arrows indicate internal void, and white arrows indicate combined void. (**B**) The total void fraction in the whole root canal filling materials. (**C**) The void fraction (internal, external, and combined) in the whole root canal filling materials. (**D**) The void volumes (apical, middle, coronal thirds). Every value is shown as mean ± SD (*n* = 10); * *p* < 0.05, ** *p* < 0.01, *** *p* < 0.001, **** *p* < 0.0001, ^ns^ *p* > 0.05.

Figure 1A shows the external void, internal void, and combined void in 3D reconstruction. The total void fraction of the modified root canal sealer was 4.316% and of iRoot SP was 3.456% (*p* < 0.05), but both were lower than EndoREZ’s 8.973% (*p* < 0.05) (Figure 1B). There was no statistically significant difference in the external void of the three sealers (*p* < 0.05). For internal void and combined void, the voids of the modified root canal sealer were not significantly different from the iRoot SP group but were lower than the EndoREZ group (Figure 1C). It suggested that the modified root canal sealer had a good sealing performance and filling quality.

Figure 1D details the percentage of void volume in the apical, middle, and coronal thirds. The voids of the modified root canal sealer in the middle and coronal thirds were 0.115 ± 0.063 and 0.280 ± 0.131 mm^3^, lower than EndoREZ’s 0.300 ± 0.162 and 0.914 ± 0.266 mm^3^ (*p* < 0.05), while close to iRoot SP’s 0.189 ± 0.261 and 0.237 ± 0.173 mm^3^ (*p* > 0.05). There was no significant difference between the void volume of three sealers in the apical third (*p* > 0.05).

### 3.2. The Dentinal Tubule Penetration

**Figure 2:**
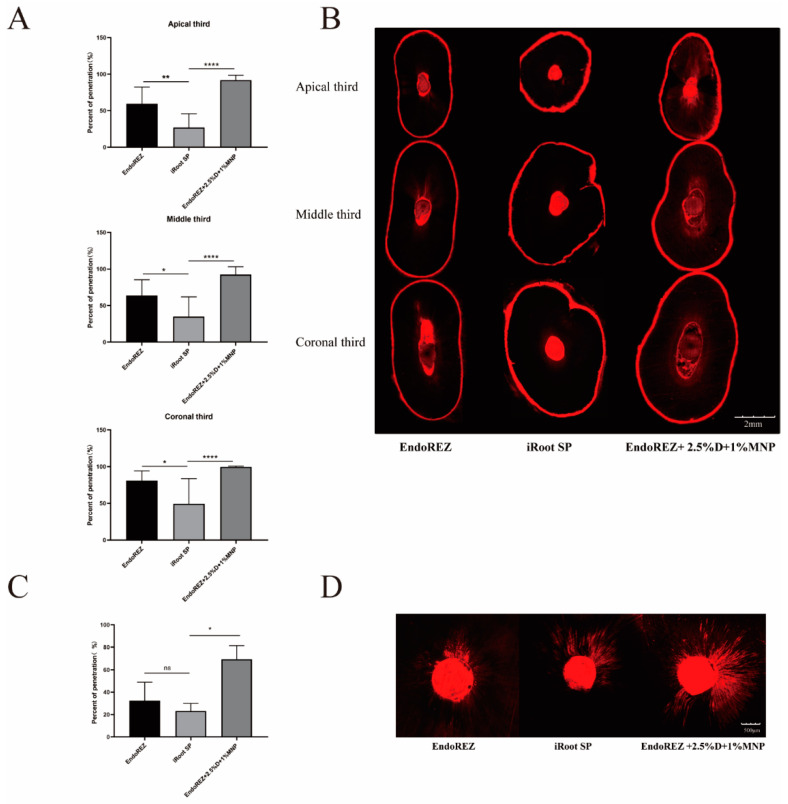
The dentinal tubule penetration of three sealers by CLSM. (**A**) Statistical analysis of dentinal tubule penetration in extracted human single-root maxillary premolars (*n* = 10) (dentinal tubule penetration = A/B—A: The portions of the canal circumference in which tubule penetration was seen were measured and added; B: the total circumference of the canal wall). (**B**) Representative images of dentinal tubule penetration in extracted human single-root maxillary premolars (4×). (**C**) Statistical analysis of dentinal tubule penetration in beagle dogs in vivo (*n* = 3). (**D**) Representative images of dentinal tubule penetration in beagle dogs in vivo (10×). Every value is shown as mean ± SD; * *p* < 0.05 ** *p* < 0.01, **** *p* < 0.0001, ^ns^ *p* > 0.05.

The penetration percentage of the modified root canal sealant was close to 100%, which was significantly higher than EndoREZ’s and iRoot’s SPs in both extracted human single-root maxillary premolars and beagle dogs in vivo (*p* < 0.05) (Figure 2). It indicated that the modified root canal sealer had a great penetration.

### 3.3. The Long-Term Antimicrobial Effects

**Figure 3:**
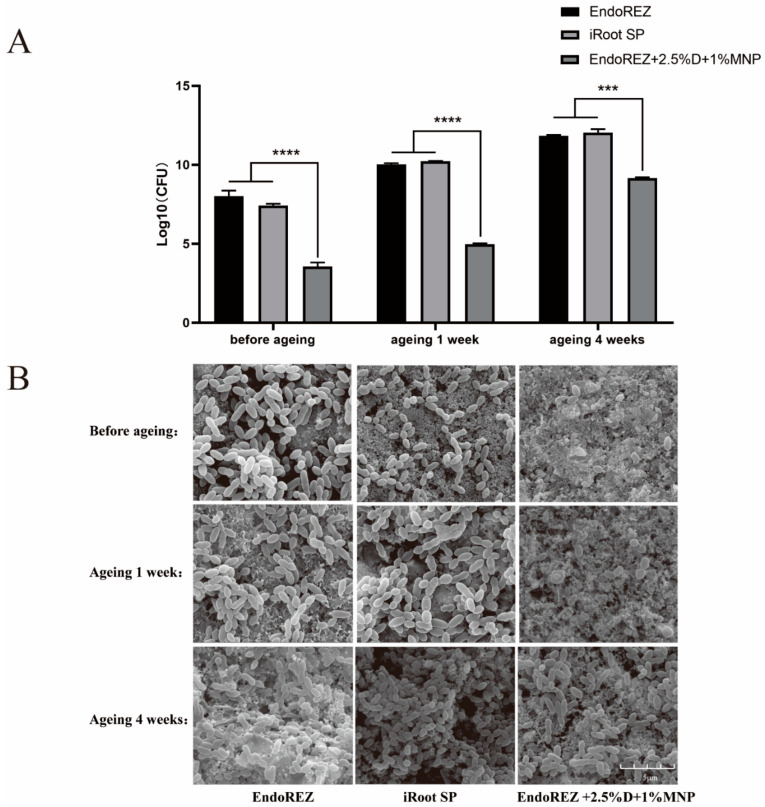
The long-term antimicrobial effects of three sealers. (**A**) Colony-forming unit counts of biofilms formed on each root canal sealer disk before aging, and after 1 and 4 weeks of aging. (**B**) Representative images of biofilms by scanning electron microscopy (SEM). Every value is shown as mean ± SD; *** *p* < 0.001, **** *p* < 0.0001.

**Figure 4:**
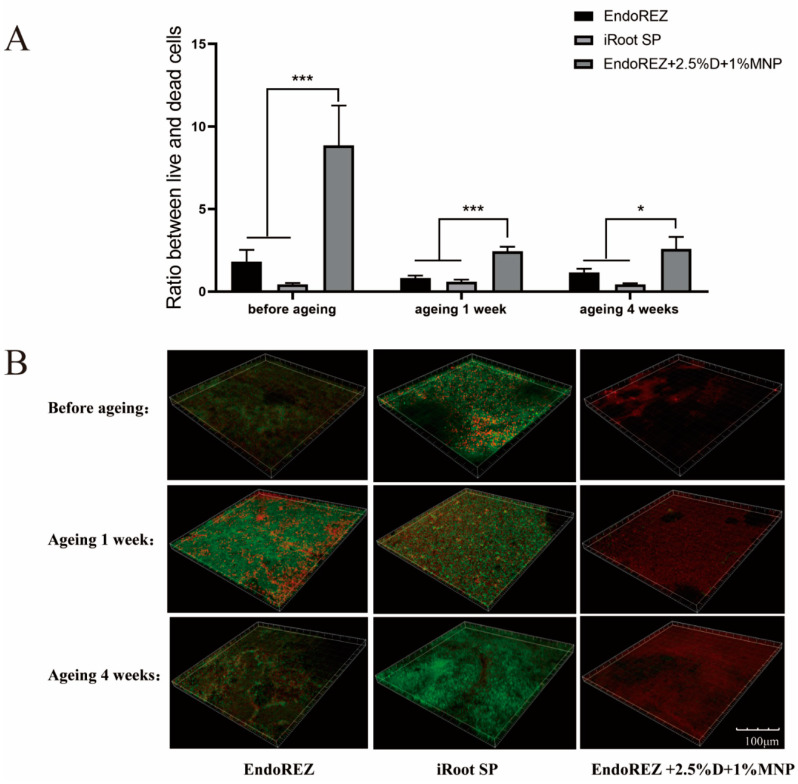
Live/dead bacteria staining. (**A**) The ratio of the live bacteria cells to dead cells was computed in line with 3 random sights of biofilms. (**B**) Representative images of biofilms (live bacteria—stained green; dead cells—stained red) in different groups. Every value is shown as mean ± SD; * *p* < 0.05, *** *p* < 0.001.

**Figure 5:**
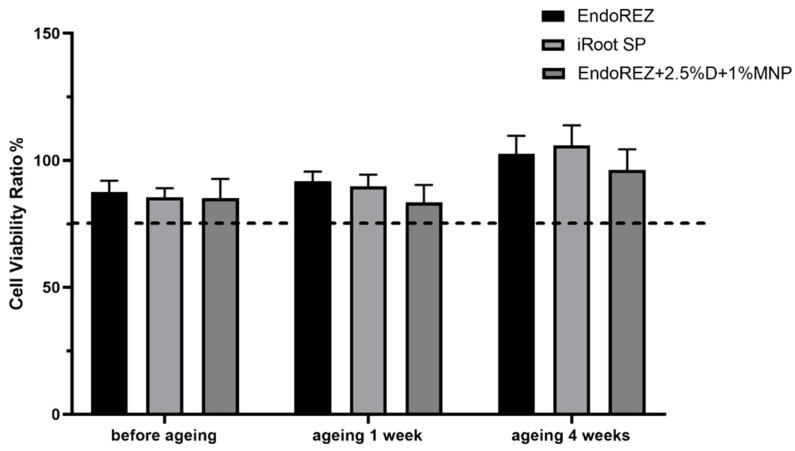
Cytotoxicity assay of sealer eluents with fibroblast (*n* = 6). Every value is shown as mean ± SD.

The CFU and bacteria numbers of the modified root canal sealer were lower than the other two sealers (*p* < 0.05) (Figure 3A,B), and the ratio between live and dead cells was much higher than the other two sealers (*p* < 0.05) (Figure 4A,B). With the extension of aging time, the antibacterial activity of the three sealers was weakened, but the modified root canal sealer always showed the best antibacterial activity, revealing it had greater long-term antibacterial activity. The cell viability ratios of the three sealers were all higher than 75% (*p* > 0.05) (Figure 5), indicating the good biocompatibility of the modified root canal sealer.

### 3.4. The Volume and Inflammatory Grade of AP

**Figure 6:**
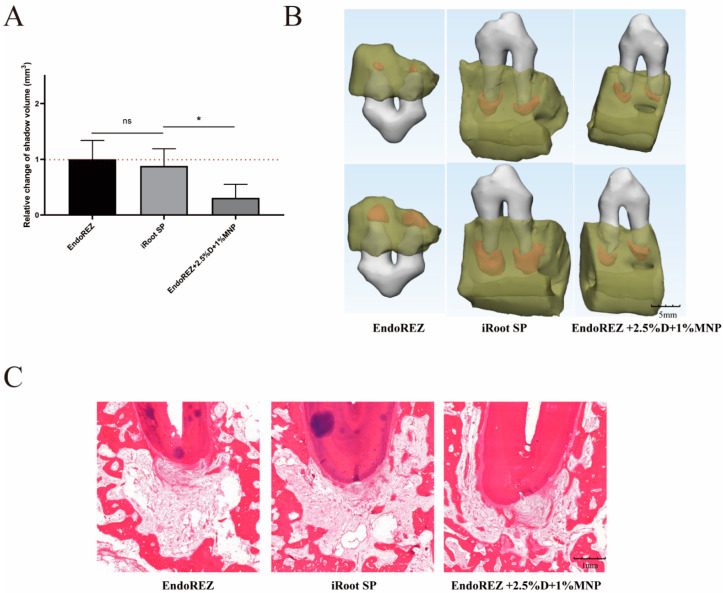
The volume and histopathological section of periapical lesions treated by three sealers. (**A**) Change in apical shadow volume after treatment with three sealers. (**B**) Change in apical shadow volume’s 3D reconstruction. (**C**) Histopathological images after treatment with three sealers. Each value is mean ± SD (*n* = 6); * *p* < 0.05, ^ns^ *p* > 0.05.

The increased volume of periapical lesions of the modified root canal sealers was smaller than the other two sealers (*p* < 0.05), while no significant difference was detected between EndoREZ and iRoot SP (*p* > 0.05) (Figure 6A,B). The histopathology indicated the modified root canal sealer had a lower inflammation degree than the other two sealers (*p* < 0.05) (Figure 6C and Table 1).

## 4. Discussion

Sealing is the major factor in the success of RCT, with 58% of treatment failures due to incomplete filling [33]. Micro-CT has been widely used in the sealing studies of root canal sealants in recent years [34,35]. As in previous studies [34], we found that all canal fillings had voids, and the apical third had fewer voids than the middle third and crown third. This difference may be due to the narrow apical region leading to a small proportion of sealers, and the lower density and diameter of dentin tubules of the apical region. The voids of external, combined, and crown third are more likely to cause root canal treatment failure due to microleakage of bacteria and their metabolites, and no significant difference was found between the modified root canal sealer and iRoot SP in these voids. No significant difference was detected between the total void fraction of the modified root canal sealer at 4.316% and iRoot SP’s 3.456%, while EndoREZ’s 8.973% was significantly lower than the others. These results indicate that MNP modified EndoREZ, which has good sealing performance, as the directional movement of MNP under the action of magnetic field reduces the bubbles in the sealer, at least as well as iRoot SP.

The penetration of the root canal sealers is one of the important criteria in measuring a sealer’s performance. Root canal sealants penetrate into dentinal tubules to form a physical barrier, enhance the contact area between root fillings and dentin, and bury residual bacteria, which can increase the overall root canal system’s sealing capacity [30]. In addition, the higher the penetration percentage of dentinal tubules, the closer the contact with the bacteria remaining in the tubules, and the more the antibacterial effect of the pore sealing agent can be exerted. CLSM with fluorescent organic dyes (such as rhodamine B) was a standard way to evaluate sealer penetration, as it is not dependent on surface quality, and no surface preparation that could cause artifacts is necessary which is superior to SEM [36,37]. However, rhodamine is a hydrophilic dye, so the sealer penetration may be overestimated especially in bioceramic sealers [38]. On the contrary, we did not find an unusually high dentin permeability in iRoot SP. We found a gradual increase in penetration from the apex to the crown in each group due to the increased number and diameter of dentinal tubules, which was consistent with previous studies [30]. Similar to our results, another methacrylate-resin-based sealer (RealSeal SE) showed a better penetration than iRoot SP [39]. EndoREZ has an extremely hydrophilic methacrylate functional group and can penetrate into dentin tubules to form micro-resin tags, which may explain this phenomenon [40]. Under the action of a magnetic field, EndoREZ modified with MNP performed best, with a penetration rate close to 100% no matter in the apex or crown, which was significantly higher than the other two groups.

Owing to the complexity of the root canal system, it is impossible to completely remove the microbes in the root canal through instrumentation, irrigation, and intracanal medication. Therefore, the antibacterial performance of root canal sealants is believed to help further reduce the number of residual microbes and eradicate infection. In our study, we observed the antibacterial activity of the three sealers was weakened with the extension of aging time, but the modified root canal sealant showed the best antibacterial activity all the time. QAS materials can bind to the cell membrane to cause bacteria lysis, thereby exerting bactericidal effects [41]. That would explain the great long-term antibacterial property of the modified root canal sealer of DMADDM.

Clinically, the successful treatment of apical periodontitis refers to the complete or partial elimination of preoperative periapical shadows without symptoms and signs [42]. However, periapical radiolucency persisted 1 year after treatment in 52%–84% of teeth [43]. In addition, CBCT transmittance greater than 1 mm was detected in 20% of successfully treated teeth on conventional periapical imaging [44]. Therefore, in our study, it is understandable that the volume of apical shadow increased after 3 months of root canal filling. The periapical lesions’ volume and histopathology indicated the modified root canal sealer had a lower degree of inflammation than the other two sealers, due to its good sealing performance and long-term antibacterial property.

Despite attempts to control for potential confounding variables, several limitations in this study design must be acknowledged. The method to evaluate dentin penetration using CLSM with rhodamine B is in doubt, as it may be overestimated in calcium–silicate-based sealers. However, this was not found in our research. We should find a better method to measure dentin penetration. In addition, because other animals (such as rats) are difficult to use in root canal filling models, we used the beagle model constructed by the early research, as it is anatomically similar to humans in terms of tooth size and root canal thickness [32]. In the beagle model, we can only use CBCT, the resolution of which is a little lower than micro-CT, due to the size of animal.

## 5. Conclusions

Overall, the current study found that compared with iRoot SP, the modified root canal sealer had good sealing performance, penetration, and long-term antibacterial property in SCT. This indicated that the modified root canal sealant, a novel antibacterial sealant used in SCT, could enter deeper into the dentin tubule and kill bacteria located deep in the dentin tubule Therefore, the novel root canal sealer might be a potential antibacterial sealer in the future clinical application, but we still need more experiments to prove its efficiency.

## Figures and Tables

**Table 1:** The histopathologic results after treatment with three sealers.

Root Canal Filing Material	The Categories of Inflammation	The Average Number of Inflammatory Cells
EndoREZ	3	281
iRoot SP	3	152
EndoREZ + 2.5% D + 1% MNP	2	95

## Data Availability

Data are contained within the article.
